# Key Factors Influencing the Operationalization and Effectiveness of Telemedicine Services in Henan Province, China: Cross-Sectional Analysis

**DOI:** 10.2196/45020

**Published:** 2024-01-05

**Authors:** Xianying He, Fangfang Cui, Minzhao Lyu, Dongxu Sun, Xu Zhang, Jinming Shi, Yinglan Zhang, Shuai Jiang, Jie Zhao

**Affiliations:** 1 National Engineering Laboratory for Internet Medical Systems and Applications The First Affiliated Hospital of Zhengzhou University Zhengzhou China; 2 School of Electrical Engineering and Telecommunications University of New South Wales Sydney Australia; 3 College of Public Health Zhengzhou University Zhengzhou China; 4 Finance Department The First Affiliated Hospital of Zhengzhou University Zhengzhou China

**Keywords:** telemedicine, service statistics, efficiency, quality management

## Abstract

**Background:**

Telemedicine has demonstrated its potential in alleviating the unbalanced distribution of medical resources across different regions. Henan, a province in China with a population of approximately 100 million, is especially affected by a health care divide. The province has taken a proactive step by establishing a regional collaborative platform for telemedicine services provided by top-tier provincial hospitals.

**Objective:**

We aim to identify the key factors that influence the current operationalization and effectiveness of telemedicine services in Henan province. The insights gained from this study will serve as valuable references for enhancing the efficient operation of telemedicine platforms in low- and middle-income regions.

**Methods:**

We analyzed service reports from the performance management system of telemedicine services in Henan province throughout 2020. Using descriptive statistics and graphical methods, we examined key influencing factors, such as management competency; device configuration; and hospital capability, capacity, and service efficacy, across hospitals at 2 different tiers. In addition, we used generalized linear models and multiple linear regression models to identify key operational factors that significantly affect the service volume and efficacy of 2 major telemedicine services, namely teleconsultation and tele-education.

**Results:**

Among the 89 tier 3 hospitals and 97 tier 2 hospitals connected to the collaborative telemedicine platform, 65 (73%) and 55 (57%), respectively, have established standardized management procedures for telemedicine services. As the primary delivery method for telemedicine services, 90% (80/89) of the tier 3 hospitals and 94% (91/97) of the tier 2 hospitals host videoconferencing consultations through professional hardware terminals rather than generic computers. Teleconsultation is the dominant service type, with an average annual service volume per institution of 173 (IQR 37-372) and 60 (IQR 14-271) teleconsultations for tier 3 and tier 2 hospitals, respectively. Key factors influencing the service volume at each hospital include available funding, management competency, the number of connected upper tiers, and the number of professional staff. After receiving teleconsultations from tier 3 (65/89, 73%) and tier 2 (61/97, 63%) hospitals, patients reported significant improvements in their medical conditions. In addition, we observed that service efficacy is positively influenced by management competency, financial incentives, the number of connected upper or lower tiers, and the involvement of participating medical professionals.

**Conclusions:**

Telemedicine has become increasingly popular in Henan province, with a notable focus on teleconsultation and tele-education services. Despite its popularity, many medical institutions, especially tier 2 hospitals, face challenges related to management competency. In addition to enhancing the effectiveness of existing telemedicine services, health care decision-makers in Henan province and other low- and middle-income regions should consider expanding the service categories, such as including remote emergency care and telesurgery, which have promise in addressing crucial health care needs in these regions.

## Introduction

### Background

To alleviate the unbalanced distribution of medical resources between metropolitan cities and rural or remote regions, telemedicine has emerged as a viable approach to connect experts at top-tier hospitals with underprivileged medical institutions and remote patients [1,2]. In the early stages, telemedicine services were primarily delivered via telephone and email for simple consultations [3], which proved ineffective in dealing with complex patient conditions. With the use of modern IT and telecommunication technologies, telemedicine services can now be delivered through high-speed networks, enabling high-resolution video, audio, and medical record sharing in real time for better efficiency [4,5]. Recent technological advances such as wearable technologies, telemedicine appliances, and 5G networks have further expanded the possibilities of new service genres in telemedicine. These include telemonitoring, remote ward rounds, telediagnosis, telepathology, remote emergency care, and tele-education [6-8].

China currently categorizes its medical institutions into 3 tiers. In general, tier 3 hospitals possess the most medical resources and are often located in metropolitan cities; tier 2 hospitals have fewer medical capabilities than tier 3 hospitals; and the tier 1 medical centers are only equipped for basic consultations, such as general practitioner services. Telemedicine significantly benefits patients at tier 2 and tier 1 hospitals from underprivileged regions by connecting them with experts at tier 3 hospitals. In addition, medical practitioners at lower-tier hospitals can receive training from experts at the higher-tier hospitals through regional telemedicine platforms; for instance, the provincial telemedicine platform in Henan province, as studied in this paper, linked 97.1% (99/102) of tier 3 hospitals and 89.6% (326/364) of tier 2 hospitals in the province. We note that 89 (87.3%) of the 102 tier 3 hospitals and 97 (26.6%) of the 364 tier 2 hospitals were covered in this study because sufficient data were available only for these hospitals. Such interhospital collaboration through telemedicine services plays a vital role not only in easing the unbalanced distribution of medical resources but also in making professional treatment for patients in remote regions more affordable [9,10].

Given the immense potential and benefits of telemedicine, many countries, particularly high-income ones, have embraced its practice. Telemedicine services in the United States have seen rapid development since the 1960s when NASA used a telemonitoring system to track the physiological indexes of astronauts on missions [11]. This pioneering effort paved the way for the emergence of telediagnosis and teleconsultation services in the country [12,13]. By 2013, 40% to 50% of hospitals in the United States provided telemedicine services. Likewise, Europe and countries such as Australia, Canada, and the United Kingdom have made significant strides to promote telehealth or telemedicine, primarily to serve their citizens in rural and remote areas [14-16]. Telemedicine now encompasses a broad range of services, including teleconsultation, telepathology, telediagnosis, remote emergency care, remote surgery, and tele-education, spanning across various medical disciplines [17,18].

The development of telemedicine in China commenced relatively later, following a similar pattern to many other low- and middle-income countries. Remote consultations in China commenced in the 1990s. In 1997, Shanghai Zhongshan Hospital and Anhui Fuyang People’s Hospital pioneered the launch of remote consultations [19]. Since the late 20th century, regional collaborative telemedicine platforms have been established in many provinces of China, connecting premium medical resources from top-tier hospitals to underprivileged ones [20-22]. As of 2021, in China, >29 provinces have set up regional platforms, covering >24,000 medical institutions to provide telemedicine services [23-26]. As a specific example, the collaborative telemedicine platform studied in this paper currently serves 425 tier 3 and tier 2 hospitals across Henan province. Its aim is to address the shortage of high-quality medical resources in underprivileged regions.

### Objectives

To the best of our knowledge, prior research articles have examined the effectiveness of telemedicine services delivered through regional collaborative platforms with regard to their service delivery processes [27,28]. In addition, some studies have focused on specific service types such as telepathology [29] and teleconsultation [23,26]. However, limited information is available concerning the operational factors crucial to ensuring the effective delivery of telemedicine services. Moreover, Henan province serves as a typical representative of low- and middle-income regions that actively promote telemedicine. Therefore, this paper presents a representative case study of the collaborative telemedicine platform in Henan province. The objective is to understand how various operational factors, including management competency, device configurations, institution capability, and the status of connected hospitals, affect the effectiveness of telemedicine services. The insights gained from this study offer valuable guidance to health care decision makers for the effective operation of regional collaborative telemedicine platforms not only in China but also in other low- and middle-income regions striving to bridge their health care divide through telemedicine.

## Methods

### Study Design

To understand the influence of different operational factors on the effectiveness of telemedicine services in low- and middle-income regions, this study centered on the collaborative telemedicine platform in Henan province for a representative case study. The platform, initiated by the provincial health department, is responsible for overseeing the operation of telemedicine services in the province. It directly connects hospitals in 18 cities of Henan province through web-based private networks.

To achieve our study objective, we designed a questionnaire to be completed by administrative and managerial personnel at all connected tier 3 and tier 2 hospitals. The questionnaire aimed to gather information on the operationalization and effectiveness of each telemedicine service from management personnel at the studied hospitals. The questions underwent a comprehensive review by technical engineers and medical professionals from 10 (2.4%) of the 425 connected hospitals and were revised based on the feedback provided before distribution. The questionnaire comprises 47 questions categorized into 5 general aspects, including management competency, equipment configuration, service capability, service efficacy, and development potentials. Each aspect is further delineated by a set of detailed factors with specific measures (Multimedia Appendix 1).

The questionnaire also underwent reliability testing, resulting in a Cronbach α coefficient of .875. The validity testing revealed a Kaiser-Meyer-Olkin coefficient of 0.766 and a significant Bartlett sphericity test (*P*<.001). Overall, the designed questionnaire demonstrated decent reliability and validity.

### Data Collection

**Figure 1:**
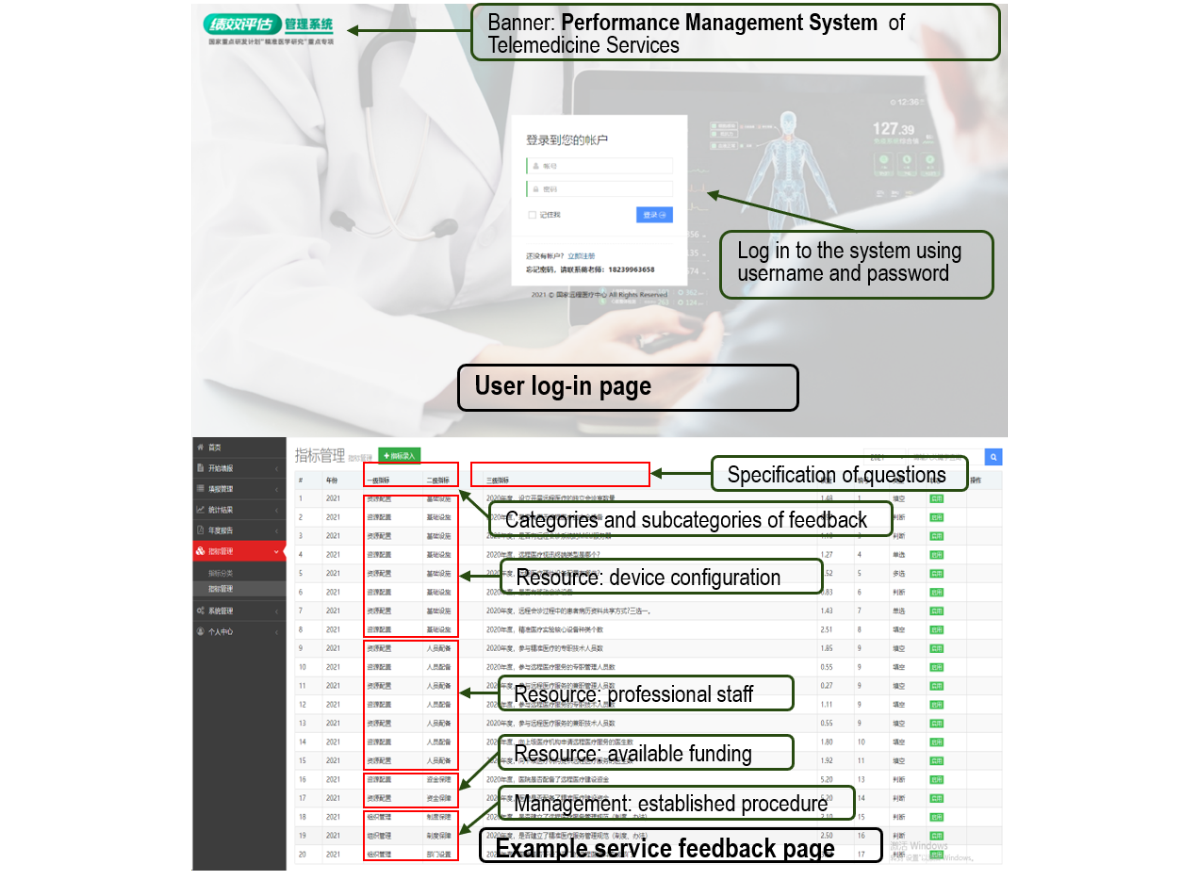
Screenshots of the performance management system of telemedicine services.

Our survey questions were integrated into the collaborative telemedicine platform as a performance management system, which collected feedback (ie, answers to our designed questions) from the corresponding administrative and managerial personnel at the hosting hospitals. Two example screenshots of the performance management system are shown in Figure 1, including the log-in page and a snippet of questions soliciting feedback. The validity of the feedback was assessed by the hosting hospitals before it was uploaded to the performance management system. Professional staff operating the system also gave regular callbacks to randomly selected respondents to verify the reliability of the feedback. The performance management system further aggregated the collected feedback per hospital, region, and year and generated evaluation reports for the respective management personnel to review. This paper covers the feedback collected by the system during the whole of 2020 (ie, from January 1, 2020, to December 31, 2020).

### Ethical Considerations

We obtained ethics approval for this study from the host institution, the First Affiliated Hospital of Zhengzhou University (2021-KY-1092-001). The ethics approval allows the researchers to collect operational information of telemedicine services through questionnaires administered to administrative and management personnel at the studied hospitals connected to the Henan provincial telemedicine platform. All participant personnel were formally informed about the use of the collected questionnaires and the objective of this study. Ethics approval was granted on the condition that the collected data would not reveal respondents’ identities and that only aggregated information (such as at hospital level) would be discussed in the research manuscript.

### Statistical Analysis

Completed questionnaires were collected from 189 participant hospitals in Henan province; after data preprocessing, which involved data cleansing and the removal of missing and invalid data, we obtained valid survey responses from 186 (98.4%) hospitals that provided telemedicine services during 2020. Of these 186 hospitals, 89 (47.8%) were tier 3 hospitals, and 97 (52.2%) were tier 2 hospitals. In our analysis, we used median values to represent statistical distributions and absolute counts with percentage values to describe counting data. Bar charts were plotted for the volume of different service types. We performed a chi-square test and a rank sum test using SPSS (version 23.0; IBM Corp) to compare the management competency, device configuration, and service capability of tier 2 and tier 3 hospitals. Furthermore, we used a generalized linear model that took the aforementioned operational factors (eg, management competency and device configuration) as independent variables to analyze their impacts on the service volume of various types (eg, teleconsultation and tele-education). A multiple linear regression model was used to analyze the influence of the identified operational factors on service efficacy with a significance level of .05 (ie, α=.05). We note that wrong and incomplete data were treated as missing values.

## Results

### Management Competency

Among the 186 studied hospitals that provided telemedicine services in 2020 via the collaborative platform in Henan province, 58% (52/89) of the tier 3 hospitals and 45% (44/97) of the tier 2 hospitals have dedicated departments managing telemedicine services. Moreover, in terms of professional staff, on average, tier 3 and tier 2 hospitals have 2.8 and 2.1 persons, respectively. In addition, 73% (65/89) of the tier 3 hospitals and 57% (55/97) of the tier 2 hospitals have established a standard management procedure for telemedicine services to be followed, meaning that the difference in this regard between tier 3 hospitals and tier 2 hospitals is statistically significant (*χ*^2^_1_=5.4; *P*=.02). To encourage medical practitioners to actively participate in telemedicine services as part of their daily duty, 54% (48/89) of the tier 3 hospitals offer financial incentives (eg, an additional bonus), whereas the corresponding figure for tier 2 hospitals is much lower (30/97, 31%; *χ*^2^_1_=5.4; *P*<.001). A telemedicine service is often requested by a lower-tier hospital to access the medical resources (eg, experts) available at an upper-tier hospital; therefore, a reasonable amount of revenue sharing between the 2 parties also contributes to the successful collaboration in a telemedicine service. However, we found that only 16% (14/89) of the tier 3 hospitals and 11% (11/97) of the tier 2 hospitals had revenue-sharing arrangements with other participating institutions, whereas the difference between tier 3 and tier 2 hospitals in this regard was not statistically significant (Table 1).

**Table 1:** Management competency regarding telemedicine services at tier 3 and tier 2 hospitals in Henan province in 2020.

Studied factors	Tier 3 hospitals (n=89), n (%)	Tier 2 hospitals (n=97), n (%)	Chi-square (*df*)	*P* value
Has dedicated department	52 (58)	44 (45)	3.2 (1)	.08
Has established management procedure	65 (73)	55 (57)	5.4 (1)	.02
Has financial incentives for participating medical practitioners	48 (54)	30 (31)	10.1 (1)	.001
Has revenue-sharing arrangements with participating hospitals	14 (16)	11 (11)	0.8 (1)	.38

### Configuration of Telemedicine Devices

The configuration of telemedicine devices is of vital importance in the effective delivery of service. The multipoint control unit (MCU) server is 1 of the critical devices that manage videoconferencing processes among participants in a telemedicine service. As revealed in our analysis, 48 (54%) of the 89 tier 3 hospitals have configured their MCU servers, approximately twice the number of tier 2 hospitals (24/97, 25%), with strong statistical significance (*χ^2^*_1_=16.7; *P*<.001). In addition to the MCU server, videoconferencing terminals that directly record and stream video and audio from participants are also deterministic influencing factors. Professional hardware terminals for videoconferencing are the optimal choice because they provide well-optimized performance and robustness. We observed that 90% (80/89) of the tier 3 hospitals and 94% (91/97) of the tier 2 hospitals had configured professional hardware terminals for telemedicine services, whereas the rest (tier 3 hospitals: 9/89, 10%; tier 2 hospitals: 6/97, 6%) used generic conferencing software (eg, Zoom and Tencent Meeting) on computers. Although the higher deployment rate of hardware terminals at tier 2 hospitals seems counterintuitive, after interviewing some administrative officers at these hospitals, we believe that tier 2 hospitals have more motivation and willingness with regard to purchasing such high-quality terminals because they are less complex to configure than MCUs and can directly benefit their patients who seek medical advice from experts at tier 3 hospitals (as well as create a better impression). As for the configuration of other professional telemedicine devices, 58% (52/89) of the tier 3 hospitals and 45% (44/97) of the tier 2 hospitals were equipped with digital pathology slide scanners, and 35% (31/89) of the tier 3 hospitals and 25% (24/97) of the tier 2 hospitals had professional recording equipment for tele-education. Fairly similar insights (ie, the device configuration status at tier 3 hospitals is better than that at tier 2 hospitals) can be obtained for other types of devices as listed in Table 2, all with statistical significance (*P*<.05).

**Table 2:** Configuration of telemedicine devices at tier 3 and tier 2 hospitals in Henan province in 2020.

Type of device	Tier 3 hospitals (n=89), n (%)	Tier 2 hospitals (n=97), n (%)	Chi-square (*df*)	*P* value
MCU^a^ server	48 (54)	24 (25)	16.7 (1)	<.001
Professional videoconferencing hardware	80 (90)	91 (94)	0.1 (1)	.33
Videoconferencing software on generic computer	9 (10)	6 (6)	0.1 (1)	.33
Digital pathology slide scanner	52 (58)	44 (45)	3.2 (1)	.08
Tele-ECG^b^ monitoring systems	57 (64)	40 (41)	9.7 (1)	.002
Multisource surgical information collection terminal	15 (17)	2 (2)	12.2 (1)	.001
Recording equipment	31 (35)	24 (25)	2.3 (1)	.13
Remote mobile ward-round vehicle	16 (18)	3 (3)	11.2 (1)	.001
Professional teleconsultation equipment	31 (35)	10 (10)	16.2 (1)	.001
Network security appliances	64 (72)	54 (56)	5.3 (1)	.02

^a^MCU: multipoint control unit.

^b^Tele-ECG: tele-electrocardiogram.

### Capability and Capacity of Hospitals

Now, we look at the capability of hospitals with regard to offering each type of telemedicine service. Among the tier 3 hospitals covered in this study, 94% (84/89), 75% (68/89), and 75% (68/89) offered teleconsultation, tele-education, and tele-electrocardiogram (tele-ECG), respectively. Among the tier 2 hospitals, 75% (73/97), 63% (61/97), and 53% (51/97) offered teleconsultation, tele-education, and telepathology, respectively. The capabilities of tier 2 and tier 3 hospitals with regard to other service types are depicted in Multimedia Appendix 2.

As shown in Table 3, with regard to the number of connected medical institutions for tier 3 and tier 2 hospitals, the median values for the connected upper-tier hospitals and lower-tier hospitals are 3 (IQR 2-9) and 16 (IQR 5-27), respectively, per tier 3 hospital and 2 (IQR 1-4) and 4 (IQR 0-15), respectively, per tier 2 hospital. Tier 3 hospitals have more connected low tier hospitals than tier 2 hospitals (*H*=−4.80; *P*<.001). As for the average service volume per hospital, that is, the number of services that have been delivered on average by the hospital, tier 3 hospitals have delivered 173 teleconsultation services, 4281 tele-ECG services, and 542 telediagnosis services, whereas tier 2 hospitals have delivered 60 teleconsultation services, 1065 tele-ECG services, and 51 telediagnosis services. Overall, the volume of the aforementioned 3 service types at tier 3 hospitals is significantly higher than that at tier 2 hospitals (*P*<.05). The average service volumes of telepathology at tier 3 and tier 2 hospitals are 120 and 114, respectively, with no statistical difference. Tele-education and remote emergency care have low volumes in both tiers (Table 4).

We highlight that the service volumes for each type of telemedicine service, which serve as the dependent variables, exhibit strong correlations with several influencing factors (ie, independent variables), including management competency, device configuration, and engagement of staff or medical practitioners. In this study, we further investigated how these factors affect the 2 most popular types of telemedicine services (ie, teleconsultation and tele-education). To be more specific, we took the service volumes of teleconsultation and tele-education as 2 dependent variables that are influenced by independent variables, including the tier to which a hospital belongs, available funding, and dedicated management department. A full list of independent variables is shown in Table 4. We constructed a generalized linear model using the variables defined herein. Our model was evaluated as a good fit by the omnibus test (*P*<.001).

Now, we highlight the factors that significantly affect the service volumes of teleconsultation and tele-education (Tables 4 and 5). First, having dedicated management departments and professional staff positively affects the service volume of teleconsultation (β=.70; *P*=.04 and β*=*.19; *P*=.002, respectively). Second, both the number of connected upper-tier hospitals and configured MCU servers positively influence the service volumes of both teleconsultation and tele-education (β=.04; *P*=.04 and β=.96; *P*=.005, respectively). Third, as shown in Table 5, the available funding, management procedure, and the number of participating medical practitioners are key influencing factors for the service volume of tele-education.

**Table 3:** Number of connected upper-tier and lower-tier hospitals and service volumes at tier 3 and tier 2 hospitals in Henan province in 2020.

Studied factors	Tier 3 hospitals, median (IQR)	Tier 2 hospitals, median (IQR)	*Z*	*P* value
Upper-tier hospitals, n	3 (2-9)	2 (1-4)	−1.88	.06
Lower-tier hospitals, n	16 (5-27)	4 (0-15)	−4.80	<.001
Service volume of teleconsultation, n	173 (37-372)	60 (14-271)	−2.70	.007
Service volume of tele-ECG^a^, n	4281 (294-24,812)	1065 (50-5103)	−2.16	.03
Service volume of telediagnosis, n	542 (62-9245)	51 (19-957)	−2.66	.008
Service volume of telepathology, n	120 (58-252)	114 (49-250)	0.43	.67
Service volume of tele-education, n	28 (10-60)	29 (9-50)	0.53	.59
Service volume of remote emergency care, n	5 (1-10)	18 (2-23)	975.00	.35

^a^Tele-ECG: tele-electrocardiogram.

**Table 4:** Analysis of key influencing factors for the service volume of teleconsultation using a generalized linear model.

Variable	β	SE	95% CI	Wald test	*P* value
Constant	5.00	.49	4.03 to 5.97	101.98	.001
Tier to which hospital belongs: tier 3 hospital (reference=tier 2 hospital)	−.15	.28	−.70 to .39	0.31	.58
Available funding (reference=no)	−1.12	.28	−1.68 to .57	15.62	<.001
Dedicated department (reference=no)	.70	.35	.02 to 1.39	4.12	.04
Management procedure (reference=no)	−.30	.37	−1.02 to .42	0.67	.41
Financial incentive (reference=no)	.22	.28	−.34 to .77	0.59	.44
Revenue-sharing arrangements (reference=no)	−.50	.36	−1.20 to .19	2.00	.16
Connected upper-tier hospitals	.04	.02	.01 to .07	4.05	.04
Connected lower-tier hospitals	.001	.000	.000 to .003	2.16	.14
Professional staff	.19	.06	.07 to .31	9.63	.002
MCU^a^ server (reference=no)	.96	.34	.29 to 1.64	7.82	.005
Type of videoconferencing terminals: professional hardware (reference=generic PC)	.18	.47	−.74 to 1.11	0.15	.70

^a^MCU: multipoint control unit.

**Table 5:** Analysis of key influencing factors for the service volume of tele-education using a generalized linear model.

Variable	β	SE	95% CI	Wald test	*P* value
Constant	3.15	.24	2.68 to 3.62	172.29	<.001
Tier to which hospital belongs: tier 3 hospital (reference=tier 2 hospital)	−.20	.23	−.64 to 2.42	0.79	.37
Available funding (reference=no)	−.81	.22	−1.25 to −.38	13.44	<.001
Dedicated department (reference=no)	.20	.24	−.26 to .66	0.72	.40
Management procedure (reference=no)	1.26	.23	.80 to 1.72	28.85	<.001
Financial incentive (reference=no)	.009	.21	−.390 to .410	0.00	.97
Revenue-sharing arrangements (reference=no)	.31	.30	−.28 to .91	1.08	.30
Connected upper-tier hospitals	−.02	.02	−.06 to .01	1.71	.19
Connected lower-tier hospitals	.000	.00	.000 to .001	0.42	.52
Professional staff	.07	.05	−.02 to .17	2.30	.13
Participating medical practitioners	.004	.00	.001 to .008	6.98	.008
Recording device (reference=no)	−.28	.21	−.69 to .14	1.70	.19

### Efficacy of Telemedicine Services

Administrative and managerial personnel at the hosting hospitals were encouraged to take surveys on the efficacy of each telemedicine service they provide. To measure service efficacy, we took into account the number of patients whose medical conditions improved after receiving telemedicine services. Our results showed that the service efficacy at tier 3 hospitals was 73%, whereas that at tier 2 hospitals was 63%. We constructed a multiple linear regression model to identify the operational factors (ie, independent variables as listed in the first column of Table 6) that could have significant impacts on service efficacy (ie, the dependent variable). The model was evaluated as a good fit by the omnibus test (*P*=.006).

We now discuss the factors that have a significant impact on service efficacy. As shown in the first column of Table 6, proper management procedures, established financial incentives, the number of connected upper-tier and lower-tier hospitals, and the number of participating medical practitioners are important influencing factors for the efficacy of teleconsultation services. With a proper management procedure (β=.15; *P*=.04) and financial incentives for participating staff (β=.14; *P*=.03), the efficacy could be significantly improved. However, having a higher number of participating medical practitioners may not always lead to better teleconsultation efficacy (β=−.001; *P*=.03), possibly owing to potential conflicts related to the treatment plans proposed by different participating physicians.

**Table 6:** Analysis of key influencing factors for the service efficacy of teleconsultation using a multiple linear regression model.

Variable	β	SE	95% CI	Wald test	*P* value
Constant	.46	.07	.34 to .59	49.512	<.001
Tier to which hospital belongs: tier 3 hospital (reference=tier 2 hospital)	.09	.06	−.03 to .21	2.363	.12
Available funding (reference=no)	.01	.07	−.12 to .15	0.037	.85
Dedicated department (reference=no)	−.10	.08	−.24 to .05	1.625	.20
Management procedure (reference=no)	.15	.07	.01 to .28	4.405	.04
Financial incentive (reference=no)	.14	.06	.02 to .26	4.825	.03
Revenue-sharing arrangements (reference=no)	−.11	.08	−.27 to .06	1.653	.20
Connected upper-tier hospitals	.010	.01	.004 to .024	6.952	.008
Connected lower-tier hospitals	−.000	.00	−.001 to .000	4.770	.03
Professional staff	−.03	−.01	.06 to .00	3.677	.06
Participating medical practitioners	−.001	.00	−.002 to .000	5.005	.03
Use of emerging IT technologies (reference=no)	.05	.05	−.09 to .19	0.514	.47

### Future Development

To estimate the future development of telemedicine services in Henan province, we studied the willingness of development and the level of financial investment at each hospital. We found that 58% (52/89) of the tier 3 hospitals and 49% (48/97) of the tier 2 hospitals had sufficient funding dedicated for the future development of telemedicine services; 48% (43/89) of the tier 3 hospitals and 38% (37/97) of the tier 2 hospitals have a detailed strategic plan; and 30% (27/89) of the tier 3 hospitals and 20% (19/97) of the tier 2 hospitals have started their exploration journey with emerging technologies such as 5G, virtual reality, and wearable devices in telemedicine services.

## Discussion

### Principal Findings

Given the important role of telemedicine in easing the unbalanced distribution of medical resources, it has been widely deployed in many low- and middle-income countries and regions, including China [28,30,31]. Henan province, as a representative of low- and middle-income regions in China, stands among the earliest provinces to establish regional collaborative telemedicine platforms, effectively connecting premium hospitals in metropolitan cities with underprivileged ones in remote and rural regions. In this study, we focus on analyzing service feedback from the provincial collaborative telemedicine platform. Through this analysis, we aim to identify key operational factors that significantly influence the effectiveness of telemedicine services provided by tier 3 and tier 2 hospitals. Our results serve as valuable references for future development and service improvement, particularly in low- and middle-income regions.

### Inadequate Managerial Capacity

Management incompetency is a prominent problem at many hospitals covered in this study: 42% (37/89) of the tier 3 hospitals and 54% (52/97) of the tier 2 hospitals did not have a dedicated management department for telemedicine services. Furthermore, the absence of proper management procedures is also a common problem. The average professional staff per hospital is <3 persons, which is particularly true at tier 2 hospitals. Our obtained insights highlight that although telemedicine has reached a high speed of deployment in China, many hospitals do not have a dedicated management and professional team for telemedicine services [21]. In addition, at lower-tier hospitals, technical staff with insufficient training may not be able to promptly handle emergency issues with their telemedicine devices and networks, negatively affecting service effectiveness [32]. Our results indicate that an established management procedure and the number of professional staff dedicated to telemedicine services at a hospital are critical factors that influence service volume and efficacy. Therefore, in low- and middle-income regions such as Henan province, seeking improvements in management competency and the training of professional staff are of high priority for increasing service effectiveness.

### Absence of Revenue-Sharing Arrangements

Revenue from the telemedicine sector is an important financial motivation for hospitals to deliver high-quality services to patients in remote and rural regions [33]. In the United States, 1 virtual clinical encounter could cost approximately US $120 [34]. In China, the cost of 1 virtual clinical encounter usually ranges from CNY 200 (US $27.9) to CNY 1000 (US $139.7) [22]. The revenue of each service is expected to be fairly shared by the hospitals that host patients and those that provide remote services. However, we found that a proper revenue-sharing arrangement has not been set up by most of the studied hospitals, which inevitably hinders interhospital collaborations. Therefore, establishing fair revenue-sharing arrangements among participating institutions is of vital importance in the operationalization of telemedicine services.

### Incapable Telemedicine Devices

Professional hardware videoconferencing terminals served by MCU servers are capable of providing real-time video and audio for telemedicine services with high resolution, security, reliability, and robustness, especially when serving multiple participating hospitals [35]. However, the high cost of such hardware combinations slows down their adoption at many hospitals [36-38]. A study conducted in 2018 showed that 61.3% of the tier 3 hospitals in China were equipped with hardware videoconferencing terminals for telemedicine services [21]. In this study, we found that 90% (80/89) of the tier 3 hospitals and 94% (91/97) of the tier 2 hospitals in Henan province have such hardware terminals. In addition, to enhance network security during the provision of telemedicine services, 72% (64/89) of the tier 3 hospitals and 56% (54/97) of the tier 2 hospitals have configured security appliances such as firewalls and intrusion detection systems. We suggest that hospitals with sufficient funding should consider making professional hardware videoconferencing terminals and network security appliances mandatory to guarantee the quality and reliability of their telemedicine services.

### Insufficient Coverage of Service Types

Our study shows that the popular telemedicine service types are teleconsultation, tele-education, tele-ECG, telepathology, and telediagnosis. Among them, tele-ECG has the most service volume because many lower-tier hospitals have the screening capability but not the capability to provide a diagnosis. Therefore, they prefer to request a tele-ECG service with upper-tier hospitals for a better diagnosis of patient conditions. Similar reasons are applied to telediagnosis and telepathology [29]. Other service types such as remote emergency care and remote surgery have low volumes currently, and their supporting devices have a low deployment rate at the respective hospitals. Given the fact that telemedicine services in remote surgery, remote ward round, and intensive care units are becoming mature [8,39,40], it is suggested that hospitals with sufficient funding could start trials with the emerging service types.

### Limitations

First, this paper focused on the critical factors for the operationalization of telemedicine services and their impacts on service effectiveness only at tier 3 (n=89) and tier 2 (n=97) hospitals in China’s Henan province; tier 1 medical institutions, which mostly serve rural and remote regions, have not been covered in this study owing to their currently low involvement in telemedicine. Second, owing to the limitation of a survey-based study, we are only able to cover the general aspect of management competency without examining detailed breakdowns, which may require rigorous interviews with the respective personnel and thus is left for future research. Third, our results primarily concern the major service types (eg, teleconsultation, tele-ECG, and tele-education), whereas the others (eg, remote surgery and remote emergency care) require sufficient data to be able to draw a trustable conclusion. In addition, this study does not cover how various factors affect patient satisfaction, which could be a valuable direction for future research.

### Conclusions

As a representative of low- and middle-income regions in China, Henan province has taken steps to develop its provincial collaborative telemedicine platform to encourage the sharing of medical resources among hospitals through various types of telemedicine services. Our study focused on the data collected from the platform throughout 2020, and it revealed that a majority of tier 2 and tier 3 hospitals offering telemedicine services are now equipped with professional devices for teleconsultation, telediagnosis, tele-ECG, and tele-education. Among all service types, tele-ECG stands out as the dominant service in terms of volume. However, our findings also highlighted some prominent issues. Among the main concerns are management incompetency, characterized by the absence of a dedicated management department; the lack of financial incentives for medical practitioners; and improper revenue sharing among participating hospitals. In addition, the current service capability is relatively limited, with only a small set of service types available, including teleconsultation, tele-ECG, telediagnosis, and tele-education. Hence, an important future direction is to expand the service categories at most hospitals, incorporating services such as remote surgery guidance and remote emergency care.

## Appendix

Multimedia Appendix 1 Details of information collected in this study.

Multimedia Appendix 2 Types of telemedicine services delivered by tier 3 and tier 2 hospitals in Henan province and their volume in percentage per hospital tier in 2020.

## Abbreviations

MCU: multipoint control unit
tele-ECG: tele-electrocardiogram

## Data Availability

The data used in this research are available upon individual request from the corresponding author.
