# Supplementary material for: Key Factors Influencing the Operationalization and Effectiveness of Telemedicine Services in Henan Province, China: Cross-Sectional Analysis
Source: J Med Internet Res. 2024 Jan 5;26:e45020. doi: 10.2196/45020 (PMC10799288; doi:10.2196/45020)
Supplement: Multimedia Appendix 1 [file jmir_v26i1e45020_app1.docx]

**Multimedia Appendix 1.** Coverage of information collected in this study.

| **General aspect** | **Detailed factor** | **Concrete measure** | **Additional description** |
| --- | --- | --- | --- |
| **Management competency** | **Dedicated department** | Has a dedicated department been setup for telemedicine services? | --- |
|  | **Management procedure** | Has a proper management procedure been established? | Such as administrative processes, service standards, etc. |
|  | **Financial motivating measure** | Is there financial motivating measure for participating medical practitioners? | Such as a bonus from each telemedicine service in addition to regular salaries. |
|  | **Revenue-sharing policy** | Is there revenue-sharing policy among participating medical institutes? | Has the revenue from telemedicine services been fairly distributed to each of the participating hospitals? |
|  | **Participating professionals** | Number of management/technical/medical staffs in each hospital.  Number of participating doctors from upper-tier hospitals.  Number of participating doctors from lower-tier hospitals. | --- |
|  | **Network security appliances** | Are there sufficient network security appliances in use? | Such as firewall devices, network intrusion detection systems, etc. |
| **Equipment configuration** | **Video-conferencing terminals** | Is a MCU for telemedicine services hosted in each hospital? | --- |
|  |  | What fraction of telemedicine services are delivered via professional video-conferencing hardware terminals, and software terminals on generic computers? | Professional hardware terminals are dedicated built for reliable and high-resolution video conferencing, which support standard media communication protocols such as H.323 and SIP.  Software terminals refer to video-conferencing software (e.g., Zoom) on generic computers. |
|  | **Telemedicine equipment** | What are the available telemedicine devices in each hospital? | Such as ①Pathology digital slide scanner, ②Tele-ECG monitoring systems, ③Telemedicine frontend terminal, ④Remote mobile ward-round vehicle, ⑤Recording equipment, ⑥Multi-source surgical information collection terminal, ⑦Others |
|  | **Sharing of medical records** | How are medical records being shared among participating hospitals during teleconsultation? | Such as from an interactive platform so that all participating doctors can view/edit the records; or via screensharing which is less convenient and only the host can edit the records; or other sharing methods such as organization emails. |
| **Service capability** | **Collaboration with other hospitals** | Number of connected upper-tier hospitals. | Upper-tier hospitals usually have better medical resources. |
|  |  | Number of connected lower-tier hospitals. | Lower-tier hospitals usually require help from the upper-tiers in managing patients with complex conditions. |
|  | **Available service category** | What are the available telemedicine services? | Such as ①teleconsultation ②tele-education ③remote ward round ④remote surgery ⑤telepathology ⑥telediagnosis ⑦tele-ECG ⑧others |
|  |  | What are the number of medical departments being covered by telemedicine services? | --- |
|  | **Available service category**  **Service volume** | What is the number of services for teleconsultation, tele-ECG, telepathology, telediagnosis, and tele-education. | We use annual service volume as a tangible measure. |
|  | **Service volume**  **Service feedback** | What service feedbacks have been collected? | Such as quality of consultation with upper-tier hospitals, tele-education with lower-tier hospitals, level of patient satisfaction, etc. |
| **Service efficacy** | **Satisfaction** | Has a survey of patient satisfaction been conducted? | --- |
|  | **Medical conditions of patients after services** | What are the changes in medical conditions of patients after receiving telemedicine services? | Such as the number of patients getting better, level of improvement in their medical conditions, etc. |
| **Development potentials** | **Available fundings** | How much of available fundings for telemedicine services in each hospital? | --- |
|  | **Strategic plan** | Is telemedicine a focused development direction in the strategic plan of each hospital? | --- |
|  | **Use of emerging technology** | Are emerging technologies in use/trial? | Such as the application of 5G, AI, wearable technology in telemedicine. |
