# Supplementary figures and images for: Key Factors Influencing the Operationalization and Effectiveness of Telemedicine Services in Henan Province, China: Cross-Sectional Analysis
Source: J Med Internet Res. 2024 Jan 5;26:e45020. doi: 10.2196/45020 (PMC10799288; doi:10.2196/45020)

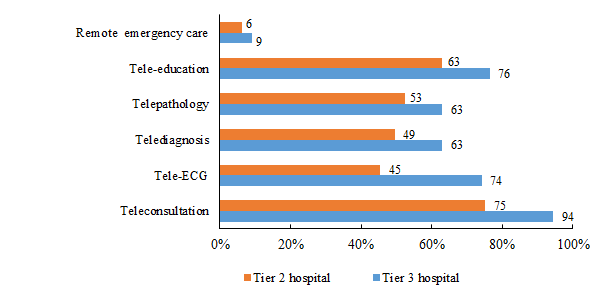

Supplement: Multimedia Appendix 2 [file jmir_v26i1e45020_app2.png]
